# The ‘myocardial work index’ does not measure myocardial work

**DOI:** 10.1093/ehjimp/qyag060

**Published:** 2026-04-28

**Authors:** David H MacIver, Henggui Zhang, Steffen E Petersen, Nay Aung, Anthony Heagerty

**Affiliations:** Biological Physics Group, Department of Astronomy and Physics, University of Manchester, Manchester, United Kingdom; Department of Cardiology, Taunton & Somerset Hospital, Somerset, United Kingdom; Biological Physics Group, Department of Astronomy and Physics, University of Manchester, Manchester, United Kingdom; William Harvey Research Institute, NIHR Barts Biomedical Research Centre, Queen Mary University London, Charterhouse Square, London EC1M 6BQ, United Kingdom; Barts Heart Centre, St Bartholomew’s Hospital, Barts Health NHS Trust, West Smithfield, London EC1A 7BE, United Kingdom; William Harvey Research Institute, NIHR Barts Biomedical Research Centre, Queen Mary University London, Charterhouse Square, London EC1M 6BQ, United Kingdom; Barts Heart Centre, St Bartholomew’s Hospital, Barts Health NHS Trust, West Smithfield, London EC1A 7BE, United Kingdom; Head of Division of Cardiovascular Sciences and Professor of Medicine, Division of Cardiovascular Sciences, Core Technology Facility (3rd floor), 46 Grafton Street, Manchester M13 9NT, United Kingdom

**This Letter refers to ‘Myocardial work analysis during semi-supine stress echocardiography: exercise response patterns in heart failure patients and controls’, by F. Sahiti**  ***et al*****., https://doi.org/10.1093/ehjimp/qyag044.**

We read with interest the report by Sahiti and colleagues. We wish to raise a fundamental concern about the physical basis of the “myocardial work” framework and propose a more rigorous and prognostically superior alternative.

The definition of mechanical work is *W* = *Fd* cos *θ*, where *F* is the applied force, *d* is the displacement through which it acts, and *θ* is the angle between force and displacement vectors. GCW, GWW, and GWI (in mmHg%) are derived from the cavity pressure and global longitudinal strain (GLS). Two independent physical objections demonstrate that this metric cannot represent myocardial work.

First, consider the vector geometry. Luminal pressure acts radially, normal to the endocardial surface. GLS occurs tangentially in the longitudinal direction. These vectors are perpendicular, giving *θ*=90° and therefore cos *θ*=0, such that *W* = *Fd*cos(90°) = 0. By the classical definition, cavity pressure performs zero work on the myocardium in the direction of its contraction. Multiplying cavity pressure by myocardial strain does not recover a non-zero work term; it produces a geometrically invalid quantity. It is not possible to measure myocardial work without the geometric information to calculate stress.

Second, the defence that MWI mirrors the pressure-volume loop whose area (mmHg·mL) represents stroke work exposes rather than resolves the problem. The pressure-volume loop correctly quantifies hydraulic work performed on blood, where cavity pressure and volume change share the same radial direction (*θ* = 0°), giving an exact energy conversion: 1 mmHg·mL = 0.133 mJ. GLS is not the displacement of blood; it is the tangential deformation of the muscle wall. The pressure-strain area conflates two physically distinct systems cavity hydraulics and myocardial mechanics without a valid physical bridge and so should not be labelled as a work index.

It may be argued that consensus endorsement supersedes dimensional precision. We respectfully disagree. Guidelines reflect evidence at a point in time and do not resolve questions of physical correctness. The empirically based LVEF has been guideline-endorsed for decades while simultaneously being described as “a measure of desperation” and subject to calls to abandon EF-based diagnostics.^1^ Clinical discriminative ability is necessary but insufficient for a measure to be legitimately interpreted as energy expenditure. The observations in the present study may be clinically important but our concern is with their physical interpretation.

Myocardial active strain energy density (MASED), also termed contractance, is defined as the area within a myocardial stress-strain loop.^2,3^ Myocardial stress (*σ*, Pa) is force per unit cross-sectional area acting on the muscle tissue itself, aligned with the direction of myocardial deformation and satisfies the directional requirement of *W* = *Fd* cos*θ* with *θ* = 0°. Integrating stress over strain then correctly recovers energy density: ∫*σdε* = J/m^3^. This is a derivation from continuum mechanics, not an analogy, and it measures what the pressure-strain area (MWI) claims to measure but cannot.

MASED's subtypes GLASED and CASED require wall thickness, chamber dimensions, and directional strain making implementation within existing workflows straightforward.^1^ We acknowledge that wall stress estimation carries geometric assumptions and that different equations yield varying absolute values.^4^ However, the quantity estimated remains energy density regardless of geometric approximation, and we have identified optimal equations for both directions.^4^ Further, with knowledge of the myocardial volume the total directional work can be calculated and allow the differentiation between pathological and physiological hypertrophy.^1,3,5,6^

In CMR cohorts with hypertension, dilated cardiomyopathy, and amyloidosis, GLASED outperformed LVEF, GLS, and pressure-strain loop indices in predicting mortality.^1^ In a second study consisting of a large community-based CMR cohort of 44 957 individuals, GLASED demonstrated the highest hazard ratio for all-cause mortality and major adverse cardiovascular events among 23 LV markers evaluated (including MWI), differentiating risk across all tertiles.^6^ Independent prospective replication is warranted and we have said so explicitly.^6^ Nevertheless, preferring a metric that fails the most basic physical test of work over one with geometric validity, physical coherence, and direct head-to-head prognostic superiority is difficult to justify.

To conclude, a measure whose force and displacement vectors are orthogonal cannot satisfy W = *Fd*cosθ and should not be labelled as myocardial work, regardless of discriminative ability or guideline endorsement. MASED is physically coherent, feasible within existing echocardiographic workflows, and prognostically superior. We invite the authors to incorporate MASED into future exercise protocols and to subject both frameworks to direct prospective comparison.

## Lead author biography


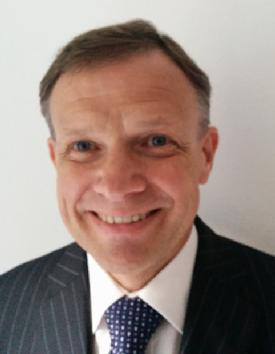


Professor David MacIver is an accomplished clinical cardiologist with over 30 years of experience, serving as Director of Echocardiography at Musgrove Park Hospital in Taunton, UK. His clinical expertise spans coronary CT, heart failure and adult congenital heart disease. Since 2014, he has held a visiting professorship with the Biological Physics Group at the University of Manchester. Professor MacIver's research focuses on the mechanisms of heart failure and the assessment of left ventricular function, integrating mathematical and computational modelling with principles of engineering physics. Over the past two decades, he has led and contributed to numerous translational and clinical studies and has published extensively in peer-reviewed journals.

## Data Availability

The data underlying this article will be shared on reasonable request to the corresponding author.
